# Synthesis, optical characterization, and antimicrobial applications of gold-coated Gd_2_O_3_:Eu^3+^ nanostructures

**DOI:** 10.1039/d5ra07449e

**Published:** 2025-11-18

**Authors:** Nguyen Thanh Huong, Pham Thi Lien, Hoang Thi Khuyen, Nguyen Thi Ngoc Anh, Do Khanh Tung, Nguyen Vu, Lam Thi Kieu Giang, Dinh Manh Tien, Nguyen Thanh Binh

**Affiliations:** a Institute of Materials Sciences, Vietnam Academy of Science and Technology 18 Hoang Quoc Viet, Nghia Do Hanoi Vietnam nthuong@ims.vast.ac.vn; b Institute of Physics, Vietnam Academy of Science and Technology 10 Dao Tan Hanoi Vietnam

## Abstract

This study reports the synthesis and characterization of Eu^3+^-doped gadolinium oxide (Gd_2_O_3_:Eu^3+^) nanomaterials and their surface-modified counterparts coated with gold nanoparticles (Au NPs). A comprehensive suite of techniques, including thermogravimetric analysis (TGA), field-emission scanning electron microscopy (FESEM), high-resolution transmission electron microscopy (HRTEM), UV-Vis absorption spectroscopy, zeta potential measurements, X-ray diffraction (XRD), Raman spectroscopy, and photoluminescence were used to analysis and evaluate the synthesized samples. The SEM, TEM and XRD results revealed the successful formation of cubic-phase Gd_2_O_3_:Eu^3+^ with uniform spherical morphology (∼80 nm) and the homogeneous deposition of sub 10 nm Au NPs on their surfaces. The photoluminescence spectra clearly displayed the characteristic Eu^3+^ emission transitions (^5^D_0_ → ^7^F_*j*_, *j* = 0–4), confirming effective doping. The introduction of Au NPs induced a surface plasmon resonance effect, which significantly enhanced the antibacterial efficacy of the nanocomposite. This was quantitatively demonstrated against a panel of clinically relevant Gram-negative and Gram-positive bacterial strains, as well as the fungal pathogen *Candida albicans* (*Escherichia coli* ATCC 25922, *Staphylococcus aureus* ATCC 25923, *Klebsiella pneumoniae* ATCC 70060, *Pseudomonas aeruginosa* ATCC 27853, and *Candida albicans* ATCC 14053). The Au-coated Gd_2_O_3_:Eu^3+^ samples exhibited significantly enhanced bactericidal efficiency compared to the uncoated ones. Our findings underscore the strategic advantages of coupling lanthanide luminescence with noble metal plasmonics, paving the way for a novel class of multifunctional nanomaterials with potent applications in antimicrobial therapy and biomedical diagnostics.

## Abstract 2

Plasmon-enhanced Gd_2_O_3_:Eu^3+^ nanostructures demonstrate strong luminescence and synergistic antibacterial performance.

## Graphical abstract

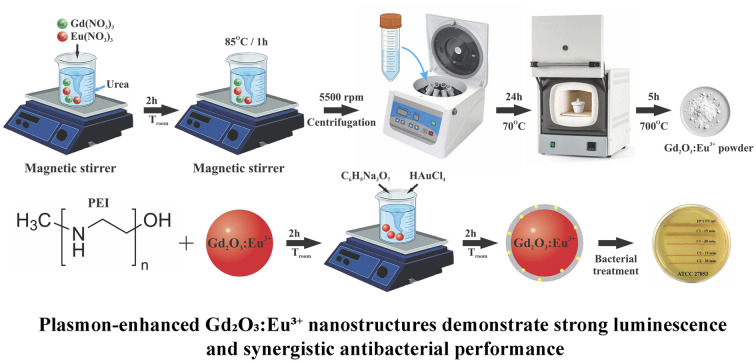

## 1 Introduction

Rare-earth-dope metal oxides represent a cornerstone of modern functional materials, with europium-doped gadolinium oxide (Gd_2_O_3_:Eu^3+^) standing out due to its exceptional luminescent properties. The Eu^3+^ ion serves as the primary emission center with characteristic red emission bands (^5^D_0_ → ^7^F_*j*_).^1–6^ The Gd_2_O_3_ host matrix is highly valued for its thermal stability, chemical inertness, and wide bandgap, which makes it particularly suitable for efficient energy transfer to the dopant Eu^3+^ ions.^7,8^ At the nanoscale, Gd_2_O_3_:Eu^3+^ NPs exhibit strong luminescence intensity with high photostability, and their optical properties can be finely tuned through morphology control and dopant concentration.^9–11^ These attributed propelled their use in diverse applications, including as: (i) phosphors in LEDs and displays; (ii) fluorescent probes in biosensing and bioimaging; (iii) fluorescent labels in biomedical applications due to their biocompatibility and sensitivity for trace-level detection.^12–16^

To further augment the functionality of these luminescent nanomaterials, the integration of plasmonic components has emerged as a powerful strategy. Decorating Gd_2_O_3_:Eu^3+^ NPs with gold (Au) NPs introduces a localized surface plasmon resonance (LSPR) effect. This LSPR can significantly amplify the local electromagnetic field around the NPs,^17,18^ which has the potential to enhance the excitation rate and modify the emission properties of the adjacent Eu^3+^ ions.^19,20^ Beyond optical modulation, Au NPs contribute their own unique bio-physicochemical properties, including strong optical absorption and scattering, and well documented antimicrobial activity.^21,22^ The biocidal mechanism of Au NPs is multifaceted, involving their attachment to microbial cell walls, which can disrupt membrane potential and permeability, interfere with vital metabolic processes, and inactivate essential enzymes, ultimately leading to microbial cell and bacteria death.

Conventional nanomaterials employed for antimicrobial applications present significant trade-offs. While Ag-based nanomaterials exhibit high intrinsic bactericidal efficacy, their widespread use is frequency constrained by concerns over poor stability in biological media and dose-dependent cytotoxicity.^21^ Similarly, photocatalytic agents like TiO_2_ and ZnO are promising but typically require energy-intensive UV or specific high-energy visible-light excitation for their activity.^21,23^ In contrast, the Gd_2_O_3_:Eu^3+^/Au hybrid developed here integrates stable red emission, plasmon-enhanced antibacterial activity, and bioimaging potential. These multifunctional properties arise from the robust and biocompatible Gd_2_O_3_ matrix coupled with the LSPR effect of Au nanoparticles,^17,18,23^ offering a simple yet effective alternative to conventional antibacterial materials.

The convergence of lanthanide luminescence and gold plasmonic thus create a novel multifunctional platform. Such a system synergistically combines the capabilities of a trackable optical label with the inherent antimicrobial action of its constituent materials. This opens avenues for developing “theragnostic” agents capable of simultaneous diagnosis and treatment. Potential applications are vast, including (i) light-activated antibacterial water purification systems; (ii) self-sanitizing medical antimicrobial surfaces; (iii) smart biosensors that can not only detect but also neutralize pathogenic microorganisms.

While the plasmon-enhanced optical properties of similar systems have been explored, a comprehensive biophysical study correlating their structural and photonic characteristics with their antimicrobial efficacy against a broad spectrum of clinically relevant pathogens remains less developed. Herein, we report the synthesis and detailed characterization of both Gd_2_O_3_:Eu^3+^ and Au-coated Gd_2_O_3_:Eu^3+^ nanomaterials. We systematically investigated their structural, colloidal and optical properties, and critically evaluated their antibacterial performance against a panel of five representative microbial strains: *Escherichia coli* (ATCC 25922), *Staphylococcus aureus* (ATCC 25923), *Klebsiella pneumoniae* (ATCC 70060), *Pseudomonas aeruginosa* (ATCC 27853), and the fungal pathogen *Candida albicans* (ATCC 14053). This work aims to establish a clear structure–property–activity relationship for these hybrid (composite) nanostructures, highlighting their significant potential for advanced biomedical and antimicrobial applications.

## 2 Experimental

### 2.1 Materials

All chemicals used in the synthesis were high-purity reagents: gadolinium(iii) nitrate hexahydrate (Gd(NO_3_)_3_·6H_2_O, 99.99%); europium(iii) nitrate pentahydrate (Eu(NO_3_)_3_·5H_2_O, 99.99%); gold(iii) chloride trihydrate (HAuCl_4_·3H_2_O, 99.9%); polyethyleneimine ((C_2_H_5_N)_*n*_, 37 wt.% in H_2_O), phosphate-buffered saline (PBS, pH 7.4) were purchased from Sigma-Aldrich; tri-sodium citrate dihydrate (C_6_H_5_Na_3_O_7_·2H_2_O), urea (CO(NH_2_)_2_ powder, 99.9%); ethanol (C_2_H_5_OH, ≥99.8%) was obtained from Merck. All synthesis procedures were carried out using deionized (DI) water.

### 2.2 Experimental

#### 2.2.1 Synthesis of Gd_2_O_3_:Eu^3+^ material

**Fig. 1:**
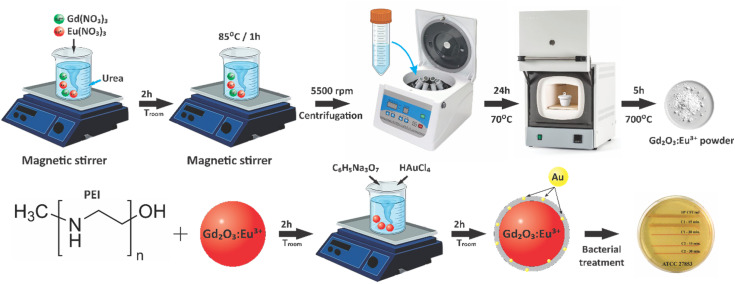
Schematic diagram of synthesis process of Gd_2_O_3_:Eu^3+^/Au *via* a wet-chemical and subsequent calcination method.

The Gd_2_O_3_:Eu^3+^ nanomaterial was synthesized *via* a wet-chemical and subsequent calcination method (Fig. 1). First, a precursor solution was prepared by mixing aqueous solution of Eu(NO_3_)_3_ (0.05 M) and Gd(NO_3_)_3_ (0.05 M) to achieve a [Eu^3+^]/[Gd^3+^] molar ratio of 5%, following by stirring for 30 min. Subsequently, this metal precursor solution was then added dropwise into 150 ml of a 0.8 M urea solution (0.8 M) contained in a reaction flask under constant stirring. The resulting mixture was stirred for 2 h at room temperature, then heated to 85 °C and maintained at this temperature for 1 h to facilitate precipitation before rapidly cooled in room temperature. The obtained product was collected by centrifugation and washed sequentially with DI water and ethanol (twice each) at 5500 rpm. The purified product was dried at 70 °C for 24 h, yielding the intermediate compound Eu^3+^-doped gadolinium carbonate hydrate (Gd(OH)CO_3_·H_2_O/Eu^3+^). The final high-purity Gd_2_O_3_:Eu^3+^ powder was obtained by calcining this intermediate in a muffle furnace at 700 °C for 5 h to remove volatile components and organic residues in order to ensure complete decomposition and crystallization.

#### 2.2.2 Synthesis of Gd_2_O_3_:Eu^3+^/Au material

The Gd_2_O_3_:Eu^3+^/Au nanocomposite was synthesized *via* an *in situ* reduction method. Briefly, 200 mg of as-synthesized Gd_2_O_3_:Eu^3+^ powder was dispersed in 20 ml of DI water *vis* ultrasonication and vortex mixing for 2 h. To functionalize the surface, 100 µl PEI was added, and the mixture was agitated for an additional 2 h. The resulting suspension was then transferred into a 100 ml round-bottom flask, and 1 ml of an aqueous HAuCl_4_ solution (0.25 M) was introduced under constant stirring. The mixture was stirred and heated to 80 °C, at which point 2 ml of a tri-sodium citrate dihydrate solution (C_6_H_5_Na_3_O_7_·2H_2_O) was added dropwise. The reaction was maintained at 80 °C for 30 min to ensure complete NPs growth. Upon cessation of heating, the mixture cooled to room temperature with continuous stirring. The final product was collected by centrifugation, washed sequentially with DI water and phosphate-buffered saline (PBS), and ultimately redispersed in PBS for storage at 4 °C to antibacterial testing.

#### 2.2.3 Application of Gd_2_O_3_:Eu^3+^/Au material in bacterial treatment

##### 2.2.3.1 Bacterial strains used in the study

The antimicrobial efficacy of the synthesized nanomaterials was assessed against five relevant microbial strains, selected to represent a diverse range of pathogenic structure and resistance mechanisms, including the Gram-negative bacteria *Escherichia coli* ATCC 25922, *Klebsiella pneumoniae* ATCC 700603, *Pseudomonas aeruginosa* ATCC 27853; Gram-positive bacteria *Staphylococcus aureus* ATCC 25923; and the pathogenic yeast *Candida albicans* ATCC 14053. All strains are standard reference organisms recommended by the Clinical and Laboratory Standards Institute (CLSI), a selection that ensured the methodological rigor, reproducibility, and a clinical comparability of the obtained results.

##### 2.2.3.2 Bacterial treatment procedure

The antibacterial activity of the synthesized nanomaterials was evaluated against the five reference strains using a streak plate method. Two sample solutions were tested: C1, a suspension of Gd_2_O_3_:Eu^3+^ (10 mg ml^−1^) and C2, a suspension of Gd_2_O_3_:Eu^3+^/Au (10 mg ml^−1^). Microbial suspensions were standardized to a concentration of 10^6^ CFU ml^−1^ in sterile saline for *Escherichia coli* ATCC 25922, *Staphylococcus aureus* ATCC 25923, *Klebsiella pneumoniae* ATCC 700603, *Pseudomonas aeruginosa* ATCC 27853, and *Candida albicans* ATCC 14053. For the assay, 100 µl of each sample (C1 or C2) was combined with 100 µl of the prepared microbial suspension in an individual sterile well. After mixing thoroughly, the mixtures were incubated for two exposure times (15 min and 30 min). For each interval, a 10 µl aliquot was collected and streaked onto the appropriate solid agar media: Blood Agar, Uri Agar, and Mueller–Hinton. The plates were then incubated, and the resulting bacterial growth along the streak lines was compared to a control (10^6^ CFU ml^−1^ suspension mixed with PBS) to determine and evaluate the bactericidal efficiency of C1 and C2.

### 2.3 Characterization of material and measurement

The structural and optical properties of the synthesized nanomaterials were characterized using a suite of analytical techniques. The crystal phase was determined by X-ray diffraction (XRD, Bruker D8 ADVANCE). Morphological analysis, including particle size and shape, was investigated using field-emission scanning electron microscopy (FESEM, Hitachi S4800) and high-resolution transmission electron microscopy (HRTEM, JEM-2100 JEOL). Thermal stability was assessed *via* thermogravimetric-differential thermal analysis (TG-DTA) on a Labsys evo 1600 system. Fourier Transform Infrared (FTIR) spectroscopy was performed on a Jasco FT/IR-6700 instrument (Jasco International, Japan). The optical characteristics were probed by UV-Vis absorption spectroscopy using a double-beam spectrophotometer, Raman spectroscopy (Horiba XploRA PLUS Raman microscope), Photoluminescence spectroscopy (Horiba Fluorolog-3 spectrofluorometer) and the fluorescence lifetime measurements were carried out using an FLS1000 Photoluminescence Spectrometer (Edinburgh Instruments, UK). Furthermore, the colloidal stability of the NPs in suspension was evaluated by Zeta potential measurements using a Zetasizer Nano ZS (Malvern, UK).

## 3 Results and discussion

### 3.1 TGA analysis

**Fig. 2:**
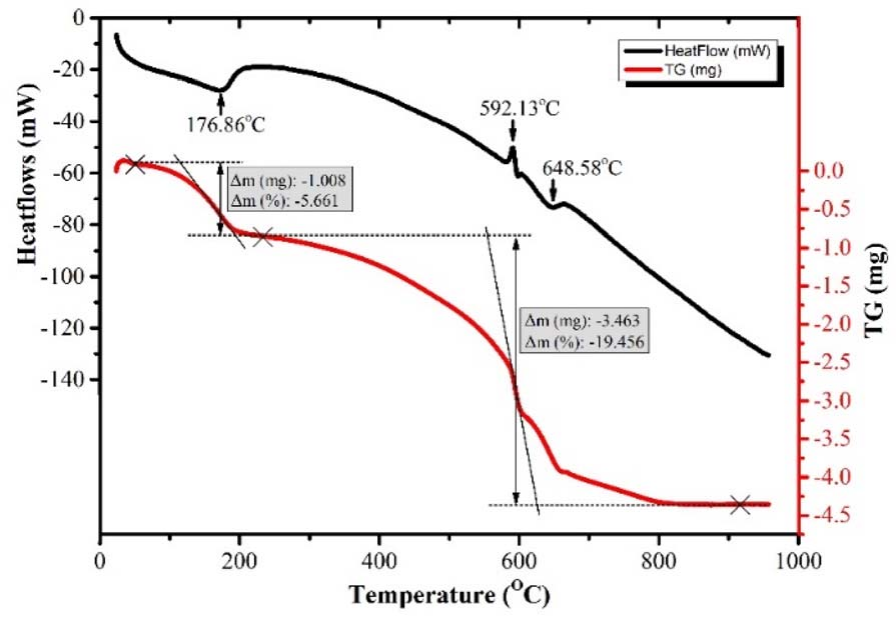
TG and DTG of Gd(OH)CO_3_·H_2_O:Eu^3+^ sample.

Thermogravimetric analysis (TGA) was employed to investigate the effect of temperature on the structural and mass changes of the Gd(OH)CO_3_·H_2_O/Eu^3+^ precursor and to determine the optimal calcination temperature. As illustrated in Fig. 2, the TGA/DTG profiles reveal a two-stage decomposition process. The first (initial) stage, exhibiting a minor mass loss of approximately 5.66% with a concomitant endothermic peak at 176.9 °C, is ascribed to the elimination of physiosorbed and crystalline water molecules. The second stage, between 250 and 650 °C, more substantial mass loss of about 19.46% occurred, which is associated with prominent DTG peaks at 592.1 °C and 648.6 °C. This stage is mainly attributed to the dehydroxylation and decarboxylation of the precursor, leading to the release of CO_2_ and H_2_O gases. The subsequent stabilization of the mass beyond 650 °C indicating the completion of decomposition process and the formation of thermally stable Gd_2_O_3_:Eu^3+^ signifies the complete conversion to the final, beyond this temperature range.

Based on this thermal profile, a calcination temperature of 700 °C was selected to guarantee complete decomposition while preventing premature sintering. The TGA data thus provide clear evidence of the sequential transformation from the metastable hydrated carbonate intermediate to the stable crystalline oxide Gd_2_O_3_:Eu^3+^ phase. This confirms that the calcination step is critical for producing a high crystalline Gd_2_O_3_:Eu^3+^ with high thermal stability for its subsequent optical functionality and potential biomedical uses.

### 3.2 Morphology characterizations

**Fig. 3:**
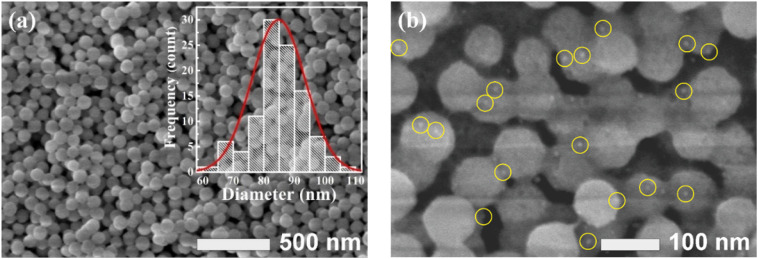
FESEM images of Gd_2_O_3_:Eu^3+^ (a) and Gd_2_O_3_:Eu^3+^/Au (b).

**Fig. 4:**
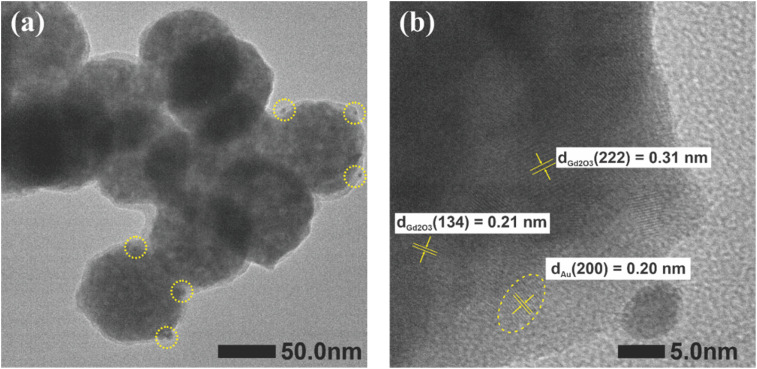
TEM (a) and HRTEM (b) images of Gd_2_O_3_:Eu^3+^/Au sample.

FESEM and HRTEM analyses were employed to characterize the morphology of the synthesized Gd_2_O_3_:Eu^3+^ and Au-coated Gd_2_O_3_:Eu^3+^ nanomaterials after calcination at 700 °C. As shown in Fig. 3a, the pristine Gd_2_O_3_:Eu^3+^ consisted of uniform, spherical particles with smooth surfaces and an average diameter of approximately 80 nm. Following the Au deposition (Fig. 3b) shows numerous smaller Au NPs (<10 nm) uniformly decorated on the surface of the Gd_2_O_3_:Eu^3+^ spheres. This Au functionalization did not disrupt the overall morphology of the host matrix but successfully created a heterostructure with intimate interfacial contact between Au and the oxide host matrix, as unequivocally by HRTEM (Fig. 4). This well-dispersed, nanoscale architecture of Au NPs on the oxide surface is critical, as it expected to enhance both light–matter interactions and facilitate the antimicrobial efficacy of the composite material.

HRTEM analysis provides nanoscale structural insight into the Au-coated Gd_2_O_3_:Eu^3+^ nanocomposites. As shown in Fig. 4a, the low-magnification image confirms the spherical morphology of Gd_2_O_3_:Eu^3+^ host particle (∼80 nm) and reveals un uniform distribution of Au NPs anchored to their surface *via* a thin polymer interlayer. The high-resolution image in Fig. 4b reveals well-defined lattice fringes, enabling crystallographic identification. Measured interplanar spacings of 0.31 nm and 0.21 nm are assigned to the (222) and (134) planes of cubic Gd_2_O_3_, respectively, while a spacing of 0.20 nm corresponds to the (200) plane of fcc Au. These *d*-spacings show excellent agreement with the XRD data, providing complementary evidence for the co-existence of both highly crystalline phases, the cubic structure of Gd_2_O_3_ and fcc Au.

The sharp lattice fringes and absence of amorphous regions further confirm the high crystallinity and structural integrity of the synthesized hetero structure with no detectable impurities or structural distortions.

**Fig. 5:**
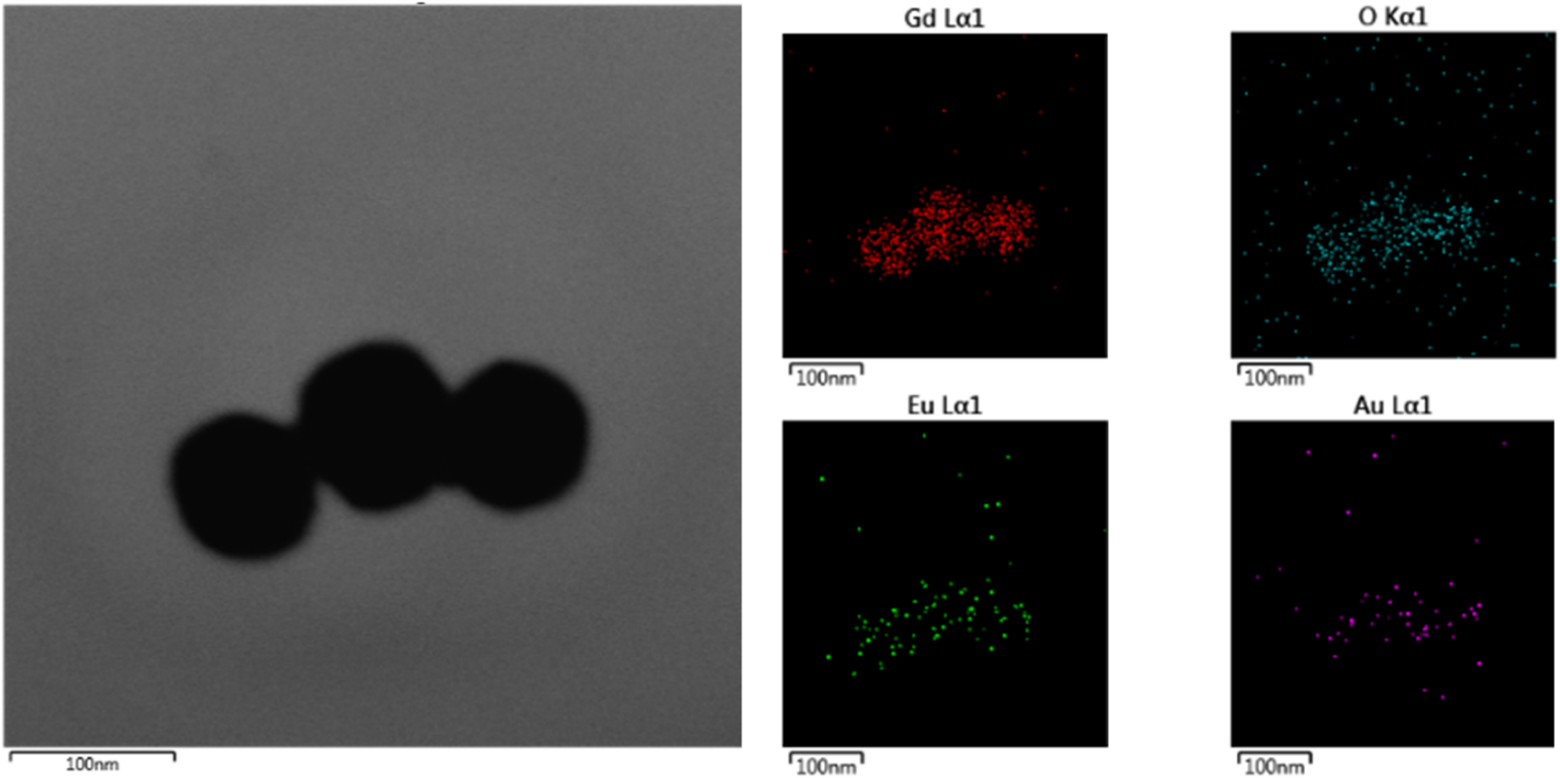
Mapping of Gd_2_O_3_:Eu^3+^/Au material.

Energy-dispersive X-ray spectroscopy (EDS) elemental mapping (Fig. 5) confirms homogeneous distribution of the constituent elements – Gd, O, Eu, and Au – within the Gd_2_O_3_:Eu^3+^/Au nanocomposite. The successful and uniform decoration of Au NPs without disrupting the host Gd_2_O_3_:Eu^3+^ matrix, signifies the formation of a stable core–shell heterostructure. This architecture provides an ideal platform for exploiting synergistic plasmonic–photonic interaction, which are anticipated to the cornerstone of its enhanced antibacterial efficacy.

### 3.3 Absorbance and zeta potential measurement results

**Fig. 6:**
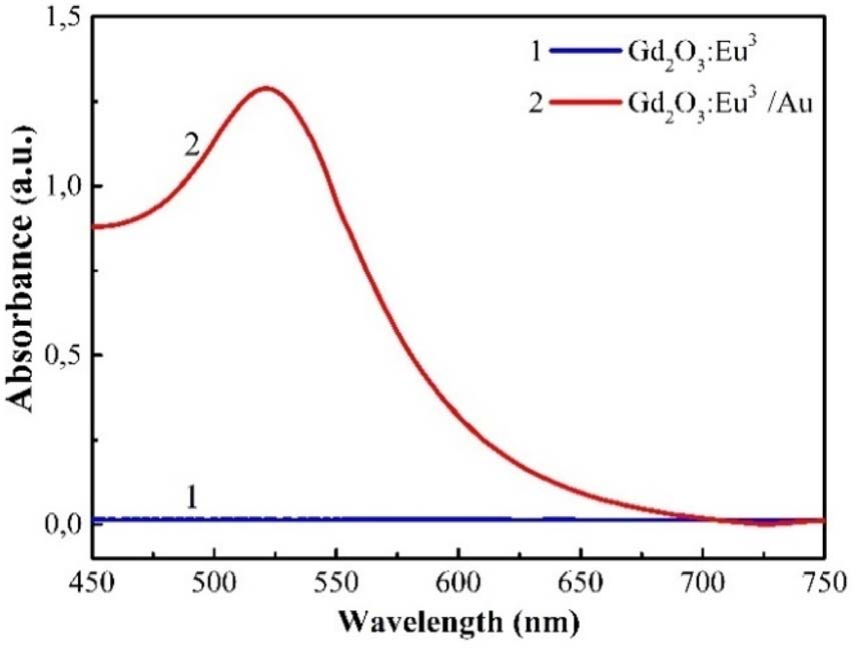
Absorption spectra of Gd_2_O_3_:Eu^3+^ and Gd_2_O_3_:Eu^3+^/Au samples.

The UV-Vis absorption spectra in Fig. 6 highlight the distinct optical properties imparted by the Au coating. Pristine Gd_2_O_3_:Eu^3+^ sample exhibits negligible absorption across the visible range (450–750 nm), which is characteristic of its wide bandgap dielectric nature of Gd_2_O_3_. In stark contrast, the Gd_2_O_3_:Eu^3+^/Au nanocomposite displays a pronounced, broad absorption band centered at approximately 525 nm. This feature is a definitive signature of the localized surface plasmon resonance (LSPR) from the deposit of Au NPs, confirming their successful integration and suggesting a potential for significant local electromagnetic field enhancement.

**Fig. 7:**
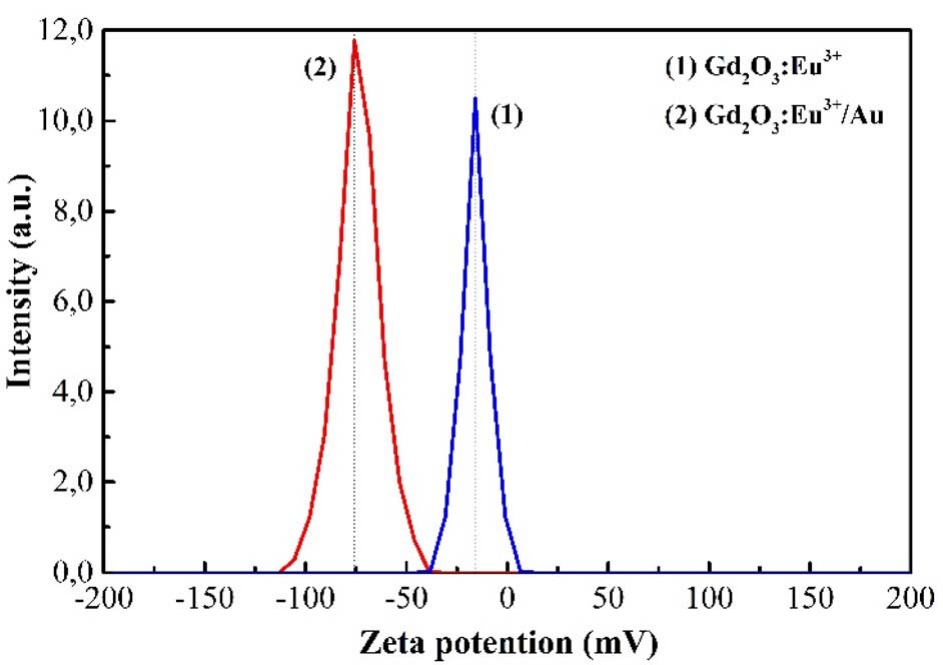
Zeta potential of Gd_2_O_3_:Eu^3+^ and Gd_2_O_3_:Eu^3+^/Au nano solution samples.

The colloidal stability of the NPs, a critical factor for biomedical applications, was quantified by zeta potential measurement (Fig. 7). The uncoated Gd_2_O_3_:Eu^3+^ NPs possessed a zeta potential of −15.8 mV, indicating moderate colloidal stability with a tendency to agglomerate. After functionalization with Au, the surface charger shifted dramatically to −74.8 mV, a value that signifies excellent electrostatic stabilization and superior dispersibility in aqueous media. This substantial increase in negative charge is attributed to the combined presence of the polymer stabilizer and the inherently negatively surface charge of the citrate-capped Au NPs.

Collectively, the emergence of a strong LSPR band and the marked shift in zeta potential provide convergent evidence for the successful and homogeneous coating of Gd_2_O_3_:Eu^3+^ with Au NPs. The enhanced colloidal stability prevents aggregation in biological media, while the plasmon properties establish the foundation for plasmon-enhanced fluorescence collectively contributing to the superior optical and antibacterial performance of the Gd_2_O_3_:Eu^3+^/Au nanocomposite.

### 3.4 X-ray diffraction results

**Fig. 8:**
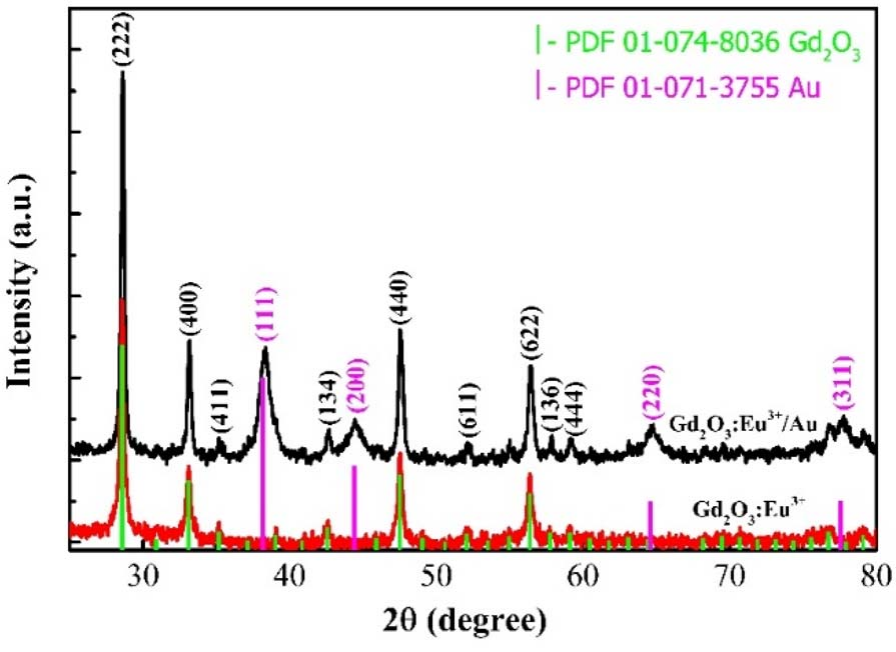
X-ray diffraction of Gd_2_O_3_:Eu^3+^ và Gd_2_O_3_:Eu^3+^/Au.

The crystalline phase and structure purity of the synthesized Gd_2_O_3_:Eu^3+^ and Gd_2_O_3_:Eu^3+^/Au nanomaterials were characterized by X-ray diffraction (XRD). As present in Fig. 8, the diffraction patterns for both samples are dominated by reflections perfectly indexed to the cubic phase of Gd_2_O_3_ (PDF 01-074-8036). For the Au-coated sample, additional distinct peaks emerge, corresponding to the (111), (200), (220) and (311) planes of fcc Au (PDF 01-071-3755). The absence of any extraneous diffraction peaks confirms the high phase purity of the synthesized products. The average crystallite size was estimated using the Scherrer equation^24^ applied to the dominant (222), (400), and (440) reflections of Gd_2_O_3_ and the (111), (200) reflections of Au. As summarized in Table 1, the calculated crystallite size of the Gd_2_O_3_ host matrix remains virtually unchanged after Au functionalization, indicating that the deposition process does not induce significant sintering or structural degradation. The observed discrepancy between the crystallite size derived from the XRD analysis and the significantly larger particle size (∼80 nm) obtained *via* HRTEM observation is a direct consequence of the material's polycrystalline nature. Specifically, the size calculated from the Scherrer equation represents the average dimension of the coherently diffracting domains (*e.g.* the constituent primary crystallites), whereas the size observed by HRTEM corresponds to the physical dimension of the secondary particles or polycrystalline aggregates – which are single entities composed of multiple smaller crystallites bonded together. This aggregation process is primarily driven by the high thermal energy input during the calcination step used to achieve the highly crystalline Gd_2_O_3_:Eu^3+^ phase and is further influenced by the subsequent surface functionalization and Au-coating processes. This structure is relevant as the overall particle size dictates cellular interaction, while the crystallite size governs the intrinsic luminescence quality.

**Table 1:** Crystalline size of Gd_2_O_3_:Eu^3+^ sample

Samples	Crystal size (nm)	
	Gd_2_O_3_	Au
Gd_2_O_3_:Eu^3+^	37.83	—
Gd_2_O_3_:Eu^3+^@Au	37.67	8.15

### 3.5 Raman measurement results

**Fig. 9:**
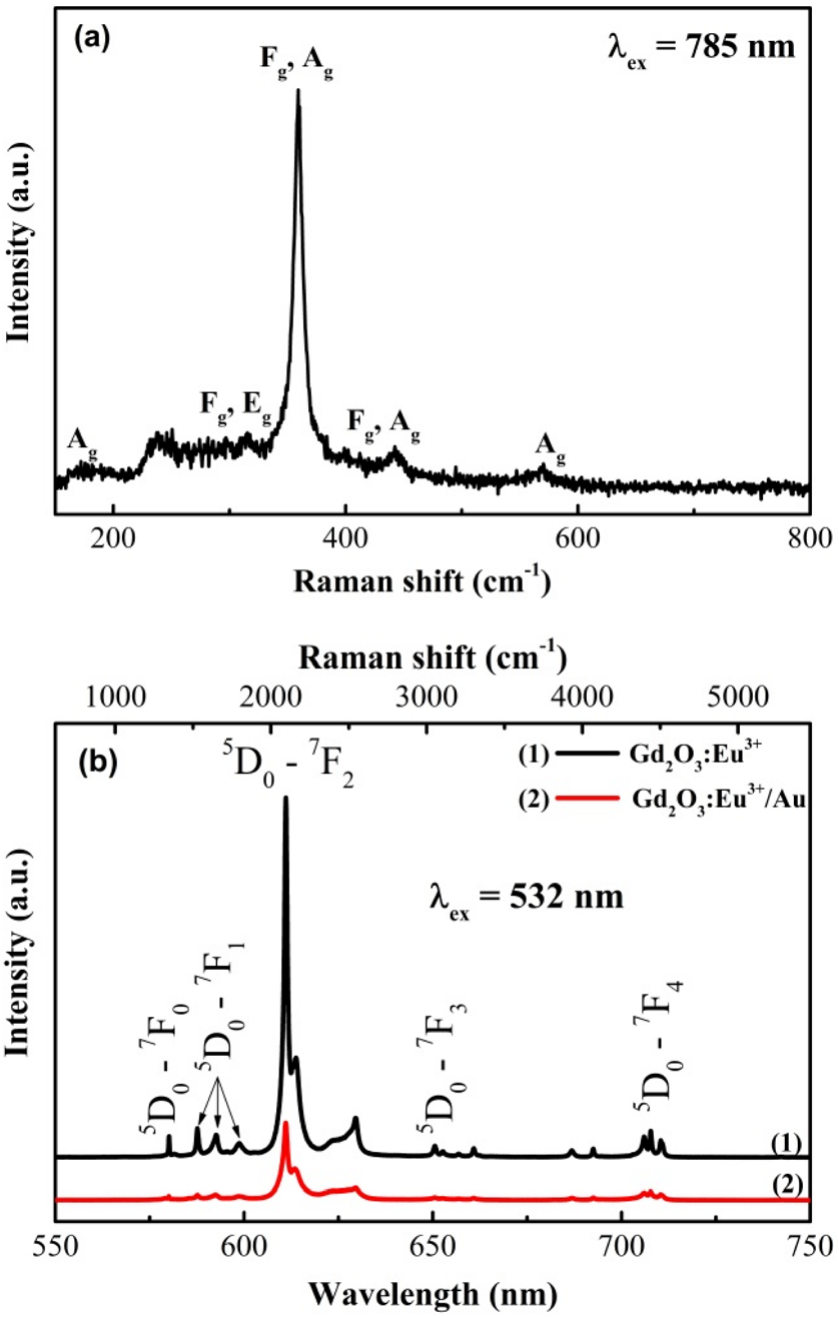
Raman spectra of (a) Gd_2_O_3_:Eu^3+^ and (b) Gd_2_O_3_:Eu^3+^/Au.

Raman spectroscopy under 785 nm excitation was employed to probe the phonon structure of the Gd_2_O_3_ lattice (Fig. 9a). The spectrum of the Gd_2_O_3_:Eu^3+^ sample displays the characteristic vibrational modes of the cubic phase. The weak low-frequency bands at ∼174 and 220 cm^−1^ (Ag) are assigned to Gd–O bending vibrations. The more prominent features at 300, 318, 360, and 440 cm^−1^(of Fg, Ag symmetry) are attributed to lattice deformation and asymmetric stretching modes. The most intense peak at 360 cm^−1^ (Fg, Ag) is identified as the symmetric Gd–O stretching vibration, a definitive signature of the cubic crystal structure of Gd_2_O_3_.^25^ A weaker band at ∼570 cm^−1^ (Ag) is associated with higher-order O–Gd–O vibrational mode. The absence of peak shift or extra modes confirms that Eu^3+^ doping does not perturb the host lattice, consistent with isovalent substitution of Gd^3+^ by Eu^3+^ ions.

Under 532 nm excitation, the spectrum is dominated by sharp intra-4f emission lines from the Eu^3+^ activator ions (Fig. 9b). The observed bands in the 580–708 nm range correspond to the ^5^D_0_ → ^7^F_*j*_ (*j* = 0–4) transitions. The pronounced intensity of the electric-dipole-dominated ^5^D_0_ → ^7^F_2_ transition, relative to the magnetic dipole ^5^D_0_ → ^7^F_1_ transition, indicates that the Eu^3+^ ions occupy lattice sites with low local inversion symmetry in the Gd_2_O_3_ lattice. In this resonant excitation regime, the efficient direct population of the Eu^3+^ 4f states results in intense luminescence that overwhelms the weaker-Raman scattering from the lattice.

A critical observation is – the significant quenching of the Eu^3+^ emission intensity in the Gd_2_O_3_:E^3+^/Au composite which is directly related to the pronounced reduction in emission intensity across the entire spectral range. This quenching effect can be explained by plasmon-induced absorption and scattering from Au NPs, together with the introduction of additional non-radiative energy transfer from the excited Eu^3+^ ions to the Au NPs and relaxation pathways, facilitated by the overlapping Au plasmon resonance. While the Au coating enhances multifunctional properties such as catalytic and antibacterial performance, competing process in this composites such as plasmon-induced scattering and the introduction of new non-radiative decay channels at the metal–oxide interface also contribute to the reduced luminescence yield. This illustrates a fundamental trade-off in such hybrid systems: the incorporation of plasmonic Au enhances functionalities like photocatalytic and antibacterial activity but at the expense of luminescence efficiency due to parasitic quenching. The design of these Gd_2_O_3_:E^3+^/Au composites much therefore be optimized to balance these opposing effects for the intended application.

### 3.6 FT-IR results

**Fig. 10:**
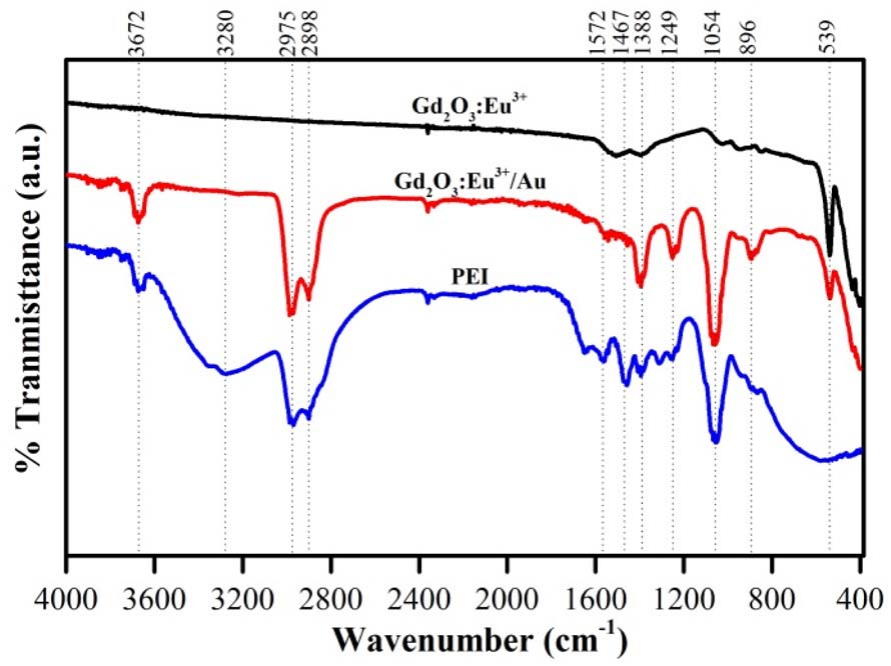
FTIR spectra of Gd_2_O_3_:Eu^3+^, Gd_2_O_3_:Eu^3+^/Au and PEI.

The FTIR spectra of PEI, Gd_2_O_3_:Eu^3+^, and the Gd_2_O_3_:Eu^3+^/Au hybrid are presented in Fig. 9. For the pristine Gd_2_O_3_:Eu^3+^ host, the strong absorption band centered around 539 cm^−1^ is characteristic of Gd–O/Eu–O stretching vibration, confirming the successful formation of the cubic lanthanide oxide lattice. The weaker bands observed at 896 and 1054 cm^−1^ are typically associated with residual C–O stretching or M–O–M bending modes within the oxide framework. In the spectrum of the final Gd_2_O_3_:Eu^3+^/Au hybrid, the appearance of new, distinct absorption peaks at 1572 cm^−1^ (–N–H bending), 1467 cm^−1^ (–CH_2_ bending), 1249 cm^−1^ and 1054 cm^−1^ (C–N stretching) provides unequivocal evidence for the successful anchoring of polyethyleneimine (PEI) onto the Gd_2_O_3_:Eu^3+^ surface prior to Au deposition. PEI thus serves as an essential intermediate layer, supplying the primary amine groups for the subsequent coordination-based anchoring of the Au NPs. Moreover, the slight shifts and changes in the relative intensity of the PEI characteristic peaks in the Au-coated sample (relative to pure PEI reference) suggest the existence of weak interactions between the amino functional groups of the PEI coating and the deposited Au NPs. Critically, the weak residual intensity of the PEI signals overall confirm the effective removal of unbound polymer during the washing process, minimizing potential cytotoxic influence and ensuring the biocompatibility necessary for antimicrobial applications (Fig. 10).

### 3.7 Photoluminescence excitation and emission properties

The photoluminescence properties of Gd_2_O_3_:Eu^3+^ and Gd_2_O_3_:Eu^3+^/Au samples were investigated through photoluminescence excitation (PLE) and emission (PL) spectroscopy.

**Fig. 11:**
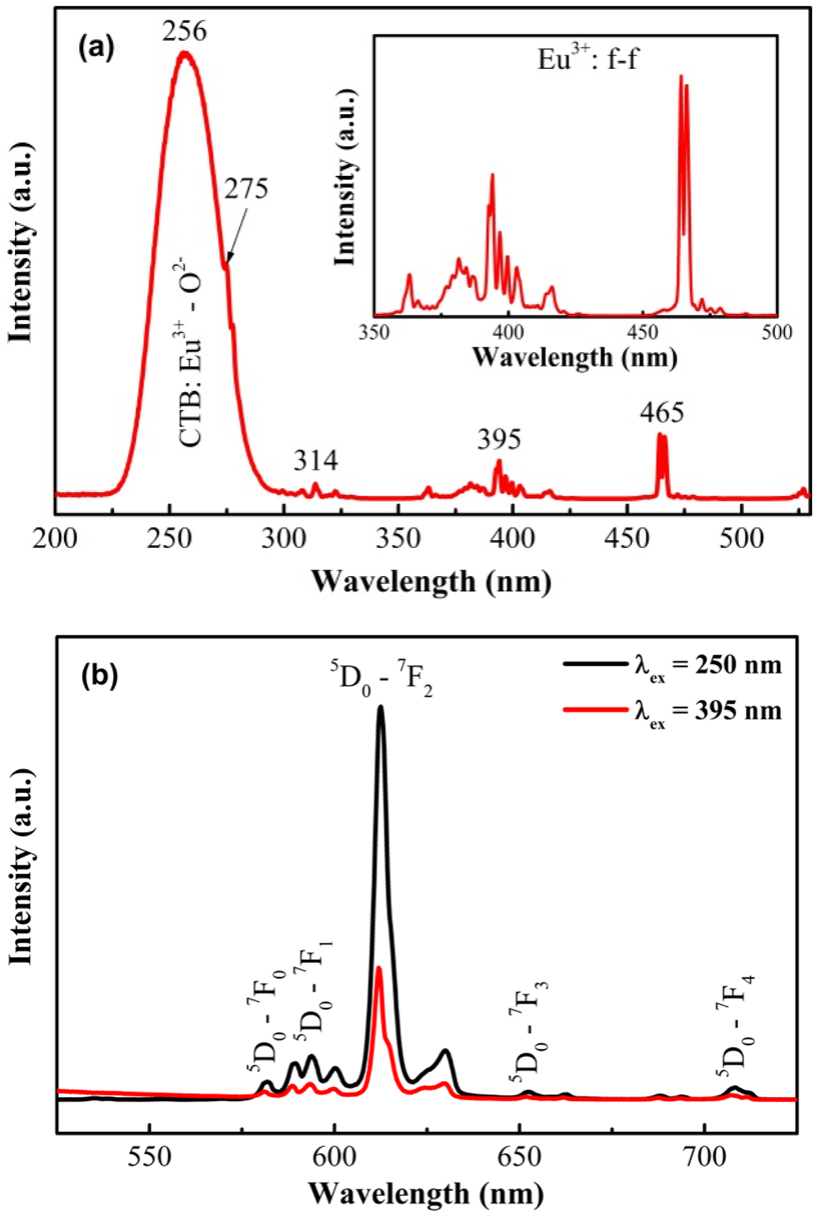
Photoluminescence spectra of Gd_2_O_3_:Eu^3+^ nanocrystals: excitation spectra (a); emission spectra (b).

The photoluminescence excitation (PLE) spectrum of Gd_2_O_3_:Eu^3+^, monitored at the dominant 611 nm emission (^5^D_0_ → ^7^F_2_), is presented in Fig. 11a. The most prominent feature is a broad, intense band centered at 256 nm (spanning 230–280 nm), which is unequivocally assigned to the charge transfer band (CTB) from the O^2−^ ligands to the Eu^3+^ center.^26^ This CTB represents the most efficient excitation pathway for populating the Eu^3+^ emitter. A series of weaker bands in the 270–320 nm range are identified as the (^8^S_7/2_ → ^6^I_*j*_) transitions of the Gd^3+^ sublattice, confirming an effective Gd^3+^ → Eu^3+^ energy transfer cascade, wherein the Gd^3+^ ions act as sensitizing antennas. Furthermore, the sharp lines in the 350–500 nm region originate from direct intra-4f excitations of Eu^3+^, with the characteristic ^7^F_0_ → ^5^L_6_ (395 nm) and ^7^F_0_ → ^5^D_2_ (465 nm) transitions being clearly resolved.^27^

The corresponding photoluminescence emission (PL) spectra under both CTB excitation (250 nm) and direct ^7^F_0_ → ^5^L_6_ excitation (395 nm) are presented in Fig. 11b. In both cases, the spectra are dominant by the characteristic red emission from the ^5^D_0_ excited state of Eu^3+^, featuring transitions to the ^7^F_*j*_ (*j* = 0–4) manifold. The emission lines are observed at approximately 580 nm (^5^D_0_ → ^7^F_0_), 592 nm (^5^D_0_ → ^7^F_1_), 611 nm (^5^D_0_ → ^7^F_2_), 650 nm (^5^D_0_ → ^7^F_3_), and 705 nm (^5^D_0_ → ^7^F_4_). The electric-dipole dominated ^5^D_0_ → ^7^F_2_ transition at 611 nm is the most intense, consistent with the Eu^3+^ ions occupying low-symmetry, non-centrosymmetric sites in the host lattice. While the overall emission intensity is significantly higher under CTB excitation due to its superior absorption cross-section, the efficient direct f–f excitation at 395 nm still produces strong red luminescence. This demonstrates the material's versatility of the Gd_2_O_3_:Eu^3+^ system, being effectively excited *via* both indirect (host sensitized) and direct activator pathways.

**Fig. 12:**
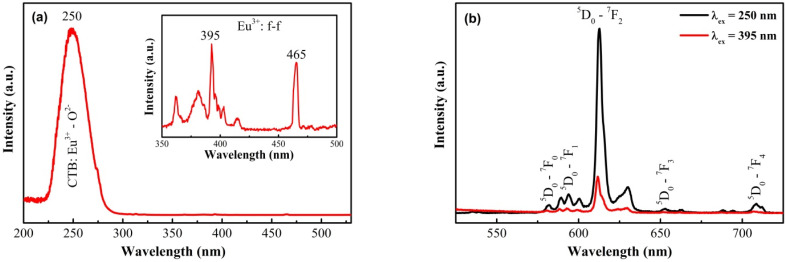
Luminescent spectra of Gd_2_O_3_:Eu^3+^/Au nanocrystals: excitation spectra (a); emission spectra (b).

Similarly, the PLE spectrum of the Gd_2_O_3_:Eu^3+^/Au nanocomposite, monitored at 611 nm (Fig. 12a), reveals that the fundamental excitation channels remain intact post-functionalization. A broad band in the 230–280 nm range, peaking at 250 nm, is assigned to the Eu^3+^–O^2−^ charge transfer band (CTB). The spectrum also retains signatures of the Gd^3+^ sensitization pathway (^8^S_7/2_ → ^6^I_*j*_ transitions in the 270–320 nm region) and characteristic intra-4f excitation of Eu^3+^, most notably the ^7^F_0_ → ^5^L_6_ (∼395 nm) and ^7^F_0_ → ^5^D_2_ (∼465 nm) transitions. This confirms the continued coexistence of CTB absorption, Gd^3+^ → Eu^3+^ energy transfer, and direct intra-4f excitations of Eu^3+^ in the hybrid system.

The corresponding emission spectra under both 250 nm (CTB) and 395 nm (f–f) excitations are shown in Fig. 12b. The characteristic ^5^D_0_ → ^7^F_*j*_ (*j* = 0–4) transitions of Eu^3+^ are clearly observed, with the electric-dipole-dominated ^5^D_0_ → ^7^F_*j*_ transition at 611 nm remaining the most intense, preserving the material's red emission profile. However, a direct comparison with the pristine material (Fig. 10b) reveals a substantial quenching of the overall luminescence intensity.

This pronounced attenuation is attributed to efficient non-radiative energy transfer from the excited Eu^3+^ ions to the Au NPs, a process driven by the spectral overlap between the Eu^3+^ emission and the Au localized surface plasmon resonance (LSPR) in the visible region (520–550 nm). The plasmonic nanostructures introduce competing decay channels, including intensified scattering and absorption, which effectively depopulate the ^5^D_0_ state and suppress radiative emission. This demonstrates a strong plasmon–fluorophore interaction between the Eu^3+^ emitting centers and the Au NPs, where the quenching mechanism dominates over any potential enhancement hence highlighting the plasmon-mediated modulation of the optical properties in such hybrid system. These findings underscore that the optical output of such hybrid systems is a sensitive function of the plasmonic architecture. By strategically engineering parameters such as the size, density, and spatial distribution of Au NPs, it may be possible to shift the balance from the observed quenching regime to a plasmon-enhanced fluorescence regime. This offers a versatile design principle for tailoring multifunctional nanomaterials for specific applications in bioimaging, sensing, or active plasmonic devices.

### 3.8 Photoluminescence excitation and emission properties

**Fig. 13:**
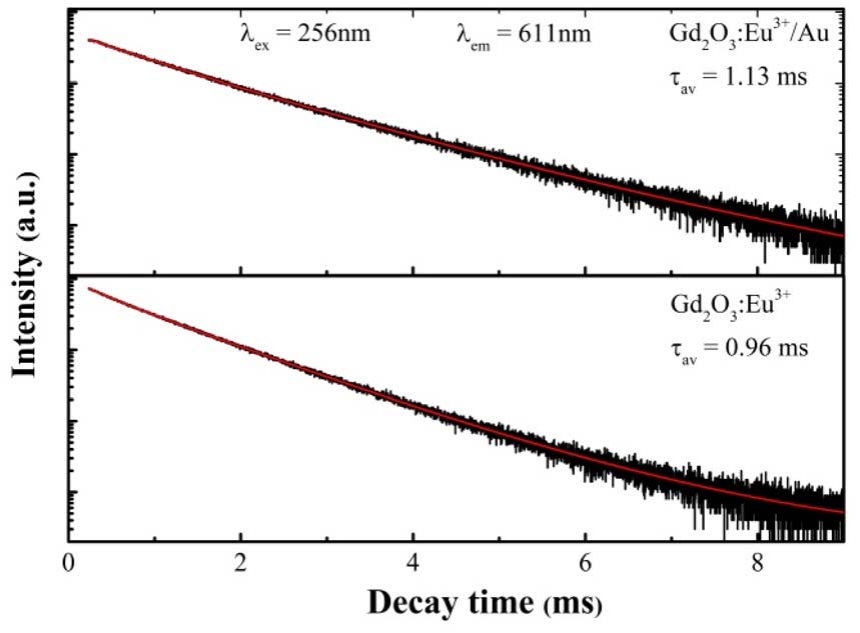
Fluorescence decay curves of Gd_2_O_3_:Eu^3+^ and Gd_2_O_3_:Eu^3+^/Au.

The fluorescence decay profiles of the Gd_2_O_3_:Eu^3+^ and Gd_2_O_3_:Eu^3+^/Au (Fig. 13) were recorded by monitoring the characteristic ^5^D_0_ → ^7^F_2_ hypersensitive transition (*λ*_em_ = 611 nm) under 256 nm excitation. The decay kinetics for both samples were accurately modelled using a bi-exponential function:*I* = *A*_1_exp(−*t*/*τ*_1_) + *A*_2_exp(−*t*/*τ*_2_)where, *I*(*t*) is the emission intensity at time *t*. *τ*_1_ and *τ*_2_ are the lifetimes of the short and long decay components, respectively, and *A*_1_, *A*_2_ are their corresponding pre-exponential factors. The average lifetime (*τ*_avg_) was then calculated using the standard formula:
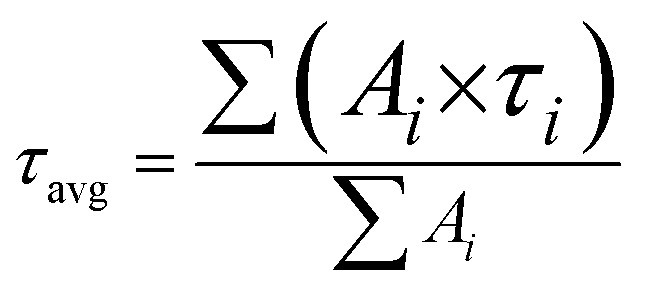


Both curves are well described by a bi-exponential function, indicating that the emission primarily arises from the radiative relaxation of the Eu^3+ 5^D_0_ level. The measured average lifetime for the Gd_2_O_3_:Eu^3+^ was determined to be 0.96 ms. Importantly, the lifetime for the Gd_2_O_3_:Eu^3+^/Au increases to 1.13 ms. This induced prolongation of the excited-state lifetime confirms that the LSPR effect of Au nanoparticles effectively couples with the Eu^3+^ emission centers. This coupling minimizes the rate of non-radiative recombination and promoted the *g* radiative decay probability of the Eu^3+^ ions. This quantitative kinetic result strongly corroborates the intensified emission observed in the steady-state photoluminescence spectra, providing definitive evidence for the plasmon-induced enhancement of luminescence efficiency in the Gd_2_O_3_:Eu^3+^/Au hybrid nanostructure (Table 2).

**Table 2:** Decay time fitting parameters and average fluorescence lifetimes of Gd_2_O_3_:Eu^3+^ and Gd_2_O_3_:Eu^3+^/Au

	Gd_2_O_3_:Eu^3+^	Gd_2_O_3_:Eu^3+^/Au
*A*_1_	2.171	2.480
*τ*_1_ (ms)	0.397	1.130
*A*_2_	7.703	2.480
*τ*_2_ (ms)	1.023	1.130
Average lifetime (ms)	**0.96**	**1.13**
Goodness of fit (*R*^2^)	0.9997	0.9990

### 3.9 Application of nanomaterial Gd_2_O_3_:Eu^3+^@Au in bacterial treatment

The antibacterial activity of Gd_2_O_3_:Eu^3+^ and Gd_2_O_3_:Eu^3+^/Au material solutions was evaluated on five representative bacterial strains: *Escherichia coli* ATCC 25922, *Staphylococcus aureus* ATCC 25923, *Klebsiella pneumoniae* ATCC 700603, *Pseudomonas aeruginosa* ATCC 27853 and *Candida albicans* ATCC 14053. The characteristics of the bacterial strains are presented in Table 3.

**Table 3:** Characteristics of 5 bacterial strains

Microorganism	ATCC code	Gram	Morphology & size	Biochemical characteristics	Research significance
*Escherichia coli*	25922	Negative	Rod-shaped, 1–3 × 0.4–0.7 µm, motile	Lactose fermentation, oxidase (−), catalase (+)	Standard Gram-negative strain for antibiotic susceptibility testing and antimicrobial evaluation
*Staphylococcus aureus*	25923	Positive	Cocci, 0.5–1.5 µm, arranged in clusters	Catalase (+), coagulase (+), mannitol fermentation	Standard Gram-positive strain, representative of pathogenic bacteria
*Klebsiella pneumoniae*	700603	Negative	Rod-shaped, 1–2 × 0.5–0.8 µm, non-motile, thick capsule	Lactose fermentation, urease (+), oxidase (−)	Gram-negative capsulated bacterium with high antibiotic resistance
*Pseudomonas aeruginosa*	27853	Negative	Rod-shaped, 1.5–3 × 0.5–0.8 µm, motile with single polar flagellum	Oxidase (+), catalase (+), produces pyocyanin pigment	Highly drug-resistant Gram-negative bacterium, common in hospital-acquired infections
*Candida albicans*	14053	— (Yeast)	Yeast cells 2–6 µm, capable of forming pseudohyphae and true hyphae	Ferments and assimilates glucose, maltose, sucrose	Pathogenic yeast strain used for antifungal activity evaluation

**Fig. 14:**
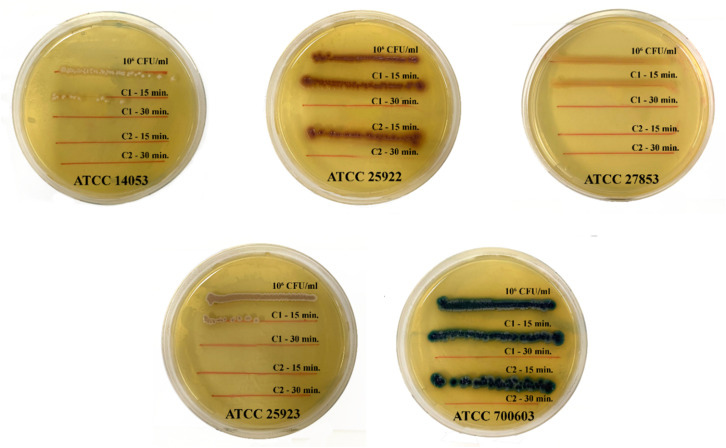
Antibacterial results of solution 1 (C1) – Gd_2_O_3_:Eu^3+^ (10 mg ml^−1^) and solution 2 (C2) – Gd_2_O_3_:Eu^3+^/Au (10 mg ml^−1^) in 15 minutes and 30 minutes.

Fig. 14 presents the bactericidal results of Gd_2_O_3_:Eu^3+^ solution (10 mg ml^−1^) and Gd_2_O_3_:Eu^3+^/Au solution (10 mg ml^−1^) for the above 5 bacterial strains. The potent broad-spectrum antimicrobial efficacy of the Au-coated Gd_2_O_3_:Eu^3+^ nanomaterials arises from a synergistic interplay between its constituent materials. The plasmonic Au shell contributes potent, intrinsic biocidal properties that target fundamental cellular structure. Upon contact, Au NPs attach to microbial surface, disrupting membrane integrity and potential. This physical compromise increases permeability, leading to ion leakage and facilitating the internalization of NPs, which can subsequently inactivate vital enzymes and disrupt metabolic pathway. This mechanism proves effective against the complex cell envelope of Gram-negative bacteria (*e.g.*, *E. coli*, *K. pneumoniae*, *P. aeruginosa*), which possess an outer membrane rich in lipopolysaccharides, the thick peptidoglycan layer of Gram-positive bacteria (*S. aureus*), and the chitinous cell wall of fungal pathogens like *C. albicans*.^28–30^

For *C. albicans*, the Au-coated nanomaterials can interact with the cell membrane and cell wall components (*e.g.*, β-glucans, mannoproteins), impairing key virulence factors, such as the adhesion proteins and morphogenetic transitions essential for it pathogenicity. Under optical excitation, the Au component significantly drives the enhanced antimicrobial activity *via* its LSPR effect. The excited plasmons can induce localized heating *via* the photothermal effect and may also promote the generation of reactive oxygen species (ROS) through plasmon-assisted photocatalytic processes.^31^ Furthermore, the Gd_2_O_3_:Eu^3+^ oxide host could contribute to this response by facilitating energy transfer or charge separation. Collectively, the combined effects of the hybrid nanostructure – membrane disruption, localized heating, and possible ROS generation – lead to a synergistic antimicrobial mechanism effective against diverse microbial strains.

## 4 Conclusions

In summary, we have successfully synthesized well-defined Gd_2_O_3_:Eu^3+^ nanospheres and their Au coated Gd_2_O_3_:Eu^3+^ nanomaterials *via* a facile multistep route. Their structural features, optical properties, and antibacterial activities were comprehensively investigated. The results reveal the cubic phase of the host matrix Gd_2_O_3_:Eu^3+^ with the homogeneous distribution of sub-10 nm Au NPs on the surface without perturbing the host crystal structure. FTIR analysis verifies the successful surface functionalization by PEI and the subsequent attachment of Au nanoparticles. The Au coating introduced a distinct localized surface plasmon resonance (LSPR), as verified by UV-Vis and Raman spectroscopy, and significantly enhanced the colloidal stability, a critical factor for biological applications. The detailed excitation, emission, and fluorescence lifetime measurements definitively established the characteristic ^5^D_0_ → ^7^F_*j*_ radiative transitions of Eu^3+^ and revealed a pronounced prolongation of the lifetime after Au deposition. This critical kinetic evidence confirms the successful plasmon-mediated modulation of the Eu^3+^ radiative pathway, enhancing the luminescence efficiency. Furthermore, *in vitro* antimicrobial assays demonstrate that the Gd_2_O_3_:Eu^3+^/Au hybrid exhibits significantly enhanced antimicrobial performance against targeted microbial strains compared to the Gd_2_O_3_:Eu^3+^ pristine host. The successful integration of Eu^3+^ luminescent centers with the Au LSPR effect yields a truly multifunctional nanomaterial that combines distinctive optical properties with superior biological activity. The observed enhancement in antimicrobial performance suggests a synergistic mechanism driven by hybrid architecture. This novel architecture holds immense promise for next generation applications in plasmon-enhanced biosensing, high-resolution bioimaging, and sophisticated light-assisted antimicrobial and theranostic treatments.

## Author contributions

All authors made significant contributions to this study. Nguyen Thanh Huong conceived the research idea, designed the experiments, supervised the project, and prepared the original draft. Pham Thi Lien carried out material synthesis and data processing. Hoang Thi Khuyen conducted antibacterial assays and interpreted the results. Nguyen Thi Ngoc Anh analysed the data and revised the manuscript. Do Khanh Tung performed structural characterizations and analysed the results. Nguyen Vu conducted optical measurements and data analysis. Lam Thi Kieu Giang performed TEM/SEM measurements and image analysis. Dinh Manh Tien carried out Raman and UV-Vis analyses and assisted with references. Nguyen Thanh Binh co-supervised the work and contributed to manuscript editing.

## Conflicts of interest

The authors declare that they have no conflicts of interest.

## Data Availability

All data are presented in the article. All data generated and analyzed during the current study are available within this manuscript.
